# Inquiry Concerning Cold Water in Gout

**Published:** 1816-03

**Authors:** William Norman

**Affiliations:** Langport, Somerset


*To the Editors of the London Medical and Physical Journal.*

GENTLEMEN,

I BEG leave, through the medium of your next Number, to request those of my professional brethren who have witnessed the topical application of cold in gouty inflammation, to favour me, at their earliest opportunity, with their observations thereon, addressed to me at Langport.

I have the honour to be,
Gentlemen,
Your very obedient servant,
WILLIAM NORMAN.

*Langport, Somerset;*

*February* 10, 1816.

 COLLEC-

